# Malocclusion among patients at Agartala, Tripura, India

**DOI:** 10.6026/973206300200258

**Published:** 2024-03-31

**Authors:** Shib Kumar Nath, Pratyaksha Singh Panwar, Kanika Singh Dhull, Pratik Surana, Ashtha Arya, Jayesh Tiwari

**Affiliations:** 1 Department of Orthodontics And Dentofacial Orthopaedics, Agartala Govt. Dental College And IGM Hospital, Agartala, Tripura, India; 2 Department of Dentistry, Government Doon Medical College, Dehradun, Uttarakhand, India; 3 Department of Pedodontics and Preventive Dentistry, Kalinga Institute of Dental Sciences, KIIT Deemed to be University, Bhubaneswar, India; 4 Department of Pedodontics and Preventive Dentistry, Maitri College of Dentistry and Research Centre, Durg, Chhattisgarh, India; 5 Department of Conservative Dentistry and Endodontics, SGT Dental College, Hospital and Research Institute, SGT University, Gurugram, Haryana - 122505, India; 6 Department of Pedodontics and Preventive Dentistry, New Horizon Dental College & Research Institute, Bilaspur, Chhattisgarh, India

**Keywords:** Malocclusion, prevalence, angle's classification

## Abstract

Malocclusion is the mal-relationship of dental arches with or without an irregularity of the teeth. Therefore, it is of interest to estimate the rate of occurrence of malocclusion within the population of Agartala city, Tripura, India. The study included 850 individuals ranging from 16 to 24 years of age, which were categorized into five distinct classifications, namely normal occlusion, Angle's Class I malocclusion, Class II Division 1, Class II Division 2, and Class III malocclusion. Data shows that normal occlusal alignment was observed in 29.41% of the participants, whereas a majority of 70.59% exhibited various forms of malocclusion among this population.

## Background:

The occlusal harmony of the dentition has pivotal implications for oral health and functionality [1]. Malocclusion, broadly defined as irregular contact between the mandibular and maxillary teeth, encompasses a diverse range of presentations with variable aetiologies including genetic, environmental, and behavioural factors. This heterogeneity often contributes to complex diagnostic and therapeutic challenges within dental practice [2]. Therefore, it is of interest to provide a comprehensive survey of the prevalence and typologies of malocclusion among a cross-sectional cohort within Agartala City, Tripura, India. This demographic investigation serves to elucidate the patterns of malocclusion within a specific geographical locale, offering insights that may enhance the scope of public health interventions and inform tailored strategies to address occlusal anomalies [1]. As malocclusion can affect masticatory performance, aesthetics, self-esteem, and speech, the investigation provides a pivotal foundation for understanding the orthodontic needs of the studied population [2]. Furthermore, due to the burgeoning interest and advancements in orthodontic technology and treatment modalities, it is vital to calibrate these innovations with the prevailing occlusal discrepancies extant within the population [3].

## Materials and Method:

## Setting and study design:

A cross-sectional observational study was conducted in Agartala City, Tripura, India to determine the prevalence and patterns of malocclusion among the local population.

## Sample size and sampling procedure:

The study sample consisted of 850 individuals, aged between 16 and 24 years. Stratified random sampling was employed to ensure representation from different age groups and genders. The participants were then grouped according to their types of occlusion based on Angle's Classification.

## Inclusion and exclusion criteria:

Individuals with permanent dentition were included in the study. Exclusion criteria comprised those with a history of orthodontic treatment or orthognathic surgery, and those suffering from syndromes affecting craniofacial morphology.

## Data collection:

Data were collected through clinical dental examinations conducted by a team of trained and calibrated dental experts. Each participant underwent a thorough dental examination to assess the type of occlusion.

## Occlusion Classification:

Occlusions were classified according to Angle's Classification into

## Group I Normal occlusion:

Normal occlusion (NO): Only those subjects were included in the study which on clinical evaluation showed bilateral Angle's Class I molar relationship with acceptable over jet and overbite and well-aligned arches.

## Group II Angle's Class I malocclusion:

Showed bilateral Angle's class I molar relationship with one or more of these characteristics: crowded incisors (Dewey type 1), protruded maxillary incisors (Dewey type 2), anterior cross-bite (Dewey type 3), unilateral or bilateral posterior cross-bite (Dewey type 4,) mesial drift of molars (Dewey type 5), anterior or posterior open bite and deep anterior overbite.

## Group III:

Class II Div 1 malocclusion

## Group IV:

Class II Div 2 malocclusion

## Group V:

Class III malocclusion

## Analysis:

The collected data was tabulated and analyzed statistically.

## Results:

The current research investigated the incidence of malocclusion in a cohort of 850 individuals, ranging in age from 16 to 24 years, within the confines of Agartala, a city located in Tripura, India. The gender distribution among the subjects was relatively balanced, with males constituting 50.33% and females comprising 49.67%, details of which are delineated in Table 1, Table 2 illustrates that a normal occlusal alignment was observed in 29.41% of the participants, whereas a majority of 70.59% exhibited various forms of malocclusion. Within this context, the prevalence of Angle's Class I malocclusion was predominant, constituting 53.41%. This was succeeded by Angle's Class II Division I and Division II malocclusions at 8.47% and 5.64%, respectively, with Angle's Class III malocclusion being the least prevalent at 3.05%.

## Discussion:

Data sheds light upon the high prevalence of malocclusion among the surveyed population in Agartala, Tripura, with a conspicuous majority, 70.59%, suffering from some form of malocclusion. This is somewhat similar to Das *et al.* who conducted an epidemiological study of malocclusion in the age group of 8-12 years in Bangalore city in 2008, and reported a high incidence of malocclusion of 71% [4].The predominance of Angle's class I malocclusion, accounting for 53.41% of cases, aligns with the literature where class I malocclusion is frequently reported as the most common form of malocclusion in various populations. However, this prevalence rate contrasts with other studies conducted within different geographical and demographic settings. This discrepancy can be attributed to genetic, environmental, and socio-economic differences which are known to influence occlusal characteristics [5-6]. The data regarding class II malocclusions, encompassing both Division 1 and Division 2, also reflect notable variance when compared to other studies. These variations warrant a more in-depth analysis into local customs, dietary habits, and healthcare accessibility, which are factors that can substantially impact dental health and occlusion [6-7]. Notably, the particularly low prevalence of Class III malocclusion at 3.05% is consistent of Trehan M *et al.* (2009) who reported prevalence of class III malocclusion of 1.4% in his study of distribution of malocclusion in Jaipur seeking orthodontic treatment [8]. Future research should incorporate a longitudinal design to monitor the development of malocclusion over time, including the evaluation of the effectiveness of early interventions. Additionally, more studies involving diverse populations could elucidate whether the data from Agartala are indicative of wider trends in malocclusion prevalence or are unique to this urban Indian demographic.

## Conclusion:

A comprehensive survey on the prevalence and typologies of malocclusion among a cross-sectional cohort within Agartala City, Tripura, India was completed. The order of prevalence is class I, followed by class II and while Class III malocclusion.

## Figures and Tables

**Table 1:** Gender wise distribution of the participants based on Angle's classification

**Occlusion**	**Male (%)**	**Female (%)**	**Total (%)**
**Normal occlusion**	**14.45**	**14.98**	**29.43%**
Angle's class I malocclusion	26.35	27.06	53.41%
Angle's class II Div 1 malocclusion	4.34	4.13	8.47%
Angle's class II Div 2 malocclusion	3.54	2.1	5.64%
Angle's class III malocclusion	1.65	1.4	3.05%
Total	50.33	49.67	100%

**Table 2:** Distribution of Sample According to occlusion classification

**Occlusion**	**N**	**%**
**Normal occlusion**	**250**	**29.43%**
Angle's class I malocclusion	454	53.41%
Angle's class II Div 1 malocclusion	72	8.47%
Angle's class II Div 2 malocclusion	48	5.64%
Angle's class III malocclusion	26	3.05
Total	850	100%
